# Adjunctive Autologous Platelet Concentrates for Periodontal Regeneration in Intrabony Defects: A Systematic Review and Meta-Analysis of Randomized Controlled Trials

**DOI:** 10.7759/cureus.109878

**Published:** 2026-05-29

**Authors:** Chadi Charrouf Abou Zeid, Flavia P Tamayo, Arturo P Jaramillo, Maria L Hattan, Luis Quintero, Meice A El Nimer Nassr

**Affiliations:** 1 Dentistry, Universidad del Zulia, Maracaibo, VEN; 2 Odontology, Central University of Venezuela, Coral Gables, USA; 3 General Practice, Universidad Estatal de Guayaquil, Machala, ECU; 4 Endodontics, Centro de Posgrado en Odontología Uninga, Bauru, BRA; 5 Dentistry, Universidad de Carabobo, Valencia, VEN; 6 General Dentistry, Tampa Medical Tower, Tampa, USA; 7 Odontology, Central University of Venezuela, Caracas, VEN

**Keywords:** blood platelets, growth factors, intrabony periodontal defects, periodontal intrabony defects, periodontitis, platelet-rich fibrin, regeneration, related autologous platelet concentrates in periodontal intrabony defect regeneration

## Abstract

Periodontal intrabony defects remain a clinical challenge because conventional periodontal therapy may reduce inflammation but does not always restore lost supporting tissues. This systematic review and meta-analysis evaluated whether autologous platelet-concentrate adjuncts improve clinical and radiographic outcomes when used with regenerative periodontal therapy. Ten randomized controlled trials were included, all assessing platelet-rich fibrin or related platelet-concentrate preparations, including concentrated growth factors, injectable platelet-rich fibrin, advanced platelet-rich fibrin, and titanium-prepared platelet-rich fibrin, in the treatment of periodontal intrabony defects. The primary clinical analysis pooled clinical attachment level or relative attachment level gain and showed a significant benefit favoring platelet-concentrate adjunct regeneration (PCA) compared with regenerative therapy without platelet concentrates. A reduced sensitivity analysis also favored the platelet-concentrate group, supporting the consistency of the clinical findings. Radiographic bone-fill or defect-depth reduction outcomes also showed a favorable direction for platelet-concentrate adjuncts, although this analysis demonstrated greater variability across studies and did not reach statistical significance. Overall, the findings suggest that autologous platelet concentrates may provide a modest but clinically relevant adjunctive benefit in periodontal intrabony-defect regeneration, particularly for clinical attachment improvement. However, radiographic outcomes should be interpreted with caution because of differences in imaging methods, grafting materials, platelet-concentrate protocols, and follow-up periods. These results support the use of platelet-concentrate adjuncts as a promising component of regenerative periodontal therapy while emphasizing the need for larger, standardized randomized trials with consistent outcome reporting and longer follow-up.

## Introduction and background

Periodontitis is a chronic inflammatory disease that progressively destroys the supporting tissues of the teeth, including the gingiva, periodontal ligament, cementum, and alveolar bone. In advanced cases, this destruction commonly results in periodontal pocket formation, clinical attachment loss, and intrabony defects, which are difficult to manage using conventional debridement alone [1-3]. Although scaling, root planing, and open-flap debridement can reduce bacterial load and inflammation, these approaches often produce periodontal repair rather than true regeneration [2,4,5]. True periodontal regeneration requires the reformation of cementum, periodontal ligament fibers, and alveolar bone in a coordinated manner, making regenerative therapy an important goal in modern periodontology [1,6-8]. Because intrabony defects provide a contained environment that may support clot stability and tissue repopulation, they have become a major focus of regenerative periodontal research [3,7-10].

In recent years, autologous platelet concentrates have gained attention as biologic adjuncts for periodontal regeneration because they may enhance wound healing, angiogenesis, and tissue maturation [1,4,6,9,11]. Platelet-rich fibrin, concentrated growth factors, injectable platelet-rich fibrin, advanced platelet-rich fibrin, and titanium-prepared platelet-rich fibrin have all been evaluated in randomized clinical trials involving periodontal intrabony defects [1-10]. These materials are attractive because they are prepared from the patient's own blood and contain a fibrin matrix enriched with platelets, leukocytes, cytokines, and growth factors [2,5,7,12]. The fibrin scaffold may help stabilize the surgical wound, while platelet-derived mediators may support fibroblast activity, osteoblast differentiation, vascular formation, and extracellular matrix deposition [3,6,8,10]. Therefore, platelet concentrates may provide both a mechanical scaffold and a biologically active environment for periodontal tissue regeneration [1,5,9,13].

Autologous platelet concentrates have been evaluated as adjuncts to several regenerative periodontal approaches, including bone grafting, open-flap debridement, and biomaterial-based therapies. Although these approaches share the goal of improving clinical attachment, periodontal pocket depth, and radiographic defect resolution, differences in platelet-concentrate preparation, defect morphology, surgical technique, and outcome reporting make the overall evidence difficult to interpret from individual trials alone [1-10,14]. Therefore, a focused systematic review and meta-analysis is needed to clarify whether platelet-concentrate adjuncts provide measurable clinical and radiographic benefits in periodontal intrabony-defect regeneration [1,4,7,10].

However, the interpretation of individual trials remains challenging because the available studies differ in surgical design, platelet-concentrate preparation, graft type, defect morphology, follow-up duration, and method of outcome assessment [1-10]. Some trials used split-mouth designs, while others used parallel-group designs, and several included relatively small sample sizes [3,6,8,10]. In addition, the control interventions were not identical across studies, as some compared platelet concentrates with open-flap debridement alone, whereas others compared platelet-concentrate combinations against bone grafts or other regenerative materials without platelet concentrates [2,4,5,7]. This methodological variation can influence the size of the reported treatment effect and may explain why some trials show stronger clinical or radiographic improvement than others [1,6,9,10,15]. For this reason, pooling comparable randomized controlled trials through meta-analysis is useful for estimating the overall effect more precisely [4,7,8].

Autologous platelet concentrates have been investigated as biologic adjuncts in periodontal regenerative therapy because they may support wound stability, growth factor release, angiogenesis, and tissue maturation. In periodontal intrabony defects, these biologic effects may be especially relevant because successful regeneration depends on both soft-tissue attachment gain and hard-tissue defect fill. However, the available evidence remains distributed across different regenerative protocols, platelet-concentrate preparations, grafting materials, and outcome-reporting methods. Therefore, a systematic review and meta-analysis is needed to synthesize the current randomized evidence and determine whether platelet-concentrate adjuncts provide measurable clinical and radiographic benefits compared with regenerative therapy without platelet concentrates [1-10,16-27].

A systematic review and meta-analysis are therefore justified because the current evidence is promising but still dispersed across different clinical protocols and outcome measurements [1-10,16-18]. By focusing on randomized controlled trials from the last decade, this review aims to reduce bias from outdated techniques and emphasize contemporary regenerative periodontal therapy [1,6,9,16,23]. The main outcomes of interest include clinical attachment level gain, relative attachment level gain, probing pocket depth reduction, and radiographic bone-fill or defect-depth reduction [2-5,7,10,20,26]. These outcomes are especially appropriate for quantitative synthesis because they are commonly reported in millimeters and can be analyzed using mean difference (MD) in RevMan 5.4 (The Cochrane Collaboration, London, UK) [1,4,8,21,27]. At the same time, risk-of-bias assessment remains essential because incomplete blinding, small sample size, unclear allocation concealment, and selective reporting may affect the certainty of the evidence [3,6,9,18,25].

Overall, autologous platelet concentrates represent a biologically plausible and clinically relevant adjunct for periodontal regeneration in intrabony defects [1-10]. Their potential benefits include improved wound stability, enhanced growth factor delivery, greater clinical attachment gain, and increased radiographic bone fill when used with regenerative surgery or grafting materials [2,5,7,9,16,20]. Nevertheless, the clinical value of these materials should be judged through a structured synthesis of randomized evidence rather than through isolated positive findings [4,8,10,21,24]. Therefore, this systematic review and meta-analysis will evaluate whether platelet-concentrate adjuncts improve periodontal regenerative outcomes compared with similar regenerative therapy without platelet concentrates, using the selected randomized controlled trials as the core evidence base [1-10,16-26].

## Review

Eligibility

Studies were considered eligible if they were randomized controlled trials evaluating periodontal regeneration in patients with periodontal intrabony defects treated with autologous platelet-concentrate adjuncts. Eligible interventions included platelet-rich fibrin, concentrated growth factors, injectable platelet-rich fibrin, advanced platelet-rich fibrin, titanium-prepared platelet-rich fibrin, or related platelet-concentrate preparations used alone or in combination with regenerative materials such as bone grafts, bioactive glass, nanohydroxyapatite, beta-tricalcium phosphate, demineralized freeze-dried bone allograft, or open-flap debridement [1-10]. The comparison group had to include periodontal regenerative therapy without a platelet-concentrate adjunct, including graft-only therapy, open-flap debridement, or another accepted regenerative control approach [1-10]. Studies were included when they reported clinical or radiographic periodontal outcomes such as clinical attachment level gain, relative attachment level gain, probing pocket depth reduction, radiographic bone-fill reduction, or radiographic defect-depth reduction [1-10]. Studies not involving periodontal intrabony defects, studies not evaluating regenerative periodontal outcomes, non-randomized studies, reviews, case reports, animal studies, and studies without usable numeric outcome data were excluded.

Study identification and search strategy

A structured literature search was performed in PubMed/MEDLINE, Embase, and the Cochrane Library/CENTRAL to identify randomized controlled trials evaluating autologous platelet-concentrate adjuncts for periodontal regeneration in intrabony defects. The search was conducted on April 25, 2026. The search was limited to human studies published in English within the previous 10 years. Reference lists of eligible studies and relevant review articles were also manually screened to identify additional randomized trials that may not have been captured by the database search (Table 1).

**Table 1:** Database-specific search strategy incorporating controlled vocabulary, free-text terms, truncation, and randomized trial filters * indicates truncation used to capture multiple word endings. MeSH: Medical Subject Headings, Emtree: Embase subject headings, CENTRAL: Cochrane Central Register of Controlled Trials, PRF: platelet-rich fibrin, CGF: concentrated growth factors, i-PRF: injectable platelet-rich fibrin, A-PRF: advanced platelet-rich fibrin, T-PRF: titanium-prepared platelet-rich fibrin, RCT: randomized controlled trial

Database	Controlled vocabulary/subject headings	Free-text keywords and truncation	Trial filter terms	Final search strategy
PubMed/MEDLINE	"Periodontitis"[MeSH]; "Periodontal Diseases"[MeSH]; "Alveolar Bone Loss"[MeSH]; "Platelet-Rich Fibrin"[MeSH]; "Blood Platelets"[MeSH]; "Growth Substances"[MeSH]; "Randomized Controlled Trial"[Publication Type]; "Clinical Trial"[Publication Type].	periodont*; "periodontal regeneration"; "periodontal defect*"; "intrabony defect*"; "intraosseous defect*"; "vertical bone defect*"; "platelet-rich fibrin"; "platelet rich fibrin"; PRF; "platelet concentrate*"; "autologous platelet concentrate*"; "concentrated growth factor*"; CGF; "injectable platelet-rich fibrin"; i-PRF; "advanced platelet-rich fibrin"; A-PRF; "titanium-prepared platelet-rich fibrin"; T-PRF.	random*; trial*; controlled; placebo; "split-mouth".	(("Periodontitis"[MeSH] OR "Periodontal Diseases"[MeSH] OR "Alveolar Bone Loss"[MeSH] OR periodont* OR "periodontal regeneration" OR "periodontal defect*" OR "intrabony defect*" OR "intraosseous defect*" OR "vertical bone defect*") AND ("Platelet-Rich Fibrin"[MeSH] OR "Blood Platelets"[MeSH] OR "Growth Substances"[MeSH] OR "platelet-rich fibrin" OR "platelet rich fibrin" OR PRF OR "platelet concentrate*" OR "autologous platelet concentrate*" OR "concentrated growth factor*" OR CGF OR "injectable platelet-rich fibrin" OR i-PRF OR "advanced platelet-rich fibrin" OR A-PRF OR "titanium-prepared platelet-rich fibrin" OR T-PRF) AND ("Randomized Controlled Trial"[Publication Type] OR "Clinical Trial"[Publication Type] OR random* OR trial* OR controlled OR placebo OR "split-mouth")).
Embase	'periodontitis'/exp; 'periodontal disease'/exp; 'alveolar bone loss'/exp; 'platelet rich fibrin'/exp; 'blood platelet'/exp; 'growth factor'/exp; 'randomized controlled trial'/exp; 'clinical trial'/exp.	periodont*; 'periodontal regeneration'; 'periodontal defect*'; 'intrabony defect*'; 'intraosseous defect*'; 'vertical bone defect*'; 'platelet-rich fibrin'; 'platelet rich fibrin'; PRF; 'platelet concentrate*'; 'autologous platelet concentrate*'; 'concentrated growth factor*'; CGF; 'injectable platelet-rich fibrin'; i-PRF; 'advanced platelet-rich fibrin'; A-PRF; 'titanium-prepared platelet-rich fibrin'; T-PRF.	random*; trial*; controlled; placebo; 'split-mouth'.	(('periodontitis'/exp OR 'periodontal disease'/exp OR 'alveolar bone loss'/exp OR periodont* OR 'periodontal regeneration' OR 'periodontal defect*' OR 'intrabony defect*' OR 'intraosseous defect*' OR 'vertical bone defect*') AND ('platelet rich fibrin'/exp OR 'blood platelet'/exp OR 'growth factor'/exp OR 'platelet-rich fibrin' OR 'platelet rich fibrin' OR PRF OR 'platelet concentrate*' OR 'autologous platelet concentrate*' OR 'concentrated growth factor*' OR CGF OR 'injectable platelet-rich fibrin' OR i-PRF OR 'advanced platelet-rich fibrin' OR A-PRF OR 'titanium-prepared platelet-rich fibrin' OR T-PRF) AND ('randomized controlled trial'/exp OR 'clinical trial'/exp OR random* OR trial* OR controlled OR placebo OR 'split-mouth')).
Cochrane Library/CENTRAL	Cochrane/CENTRAL does not use MeSH in the same way as PubMed for all searches; therefore, the strategy emphasized sensitive free-text searching.	periodont*; "periodontal regeneration"; "periodontal defect*"; "intrabony defect*"; "intraosseous defect*"; "vertical bone defect*"; "platelet-rich fibrin"; "platelet rich fibrin"; PRF; "platelet concentrate*"; "autologous platelet concentrate*"; "concentrated growth factor*"; CGF; "injectable platelet-rich fibrin"; i-PRF; "advanced platelet-rich fibrin"; A-PRF; "titanium-prepared platelet-rich fibrin"; T-PRF.	random*; trial*; controlled; "split-mouth".	((periodont* OR "periodontal regeneration" OR "periodontal defect*" OR "intrabony defect*" OR "intraosseous defect*" OR "vertical bone defect*") AND ("platelet-rich fibrin" OR "platelet rich fibrin" OR PRF OR "platelet concentrate*" OR "autologous platelet concentrate*" OR "concentrated growth factor*" OR CGF OR "injectable platelet-rich fibrin" OR i-PRF OR "advanced platelet-rich fibrin" OR A-PRF OR "titanium-prepared platelet-rich fibrin" OR T-PRF) AND (random* OR trial* OR controlled OR "split-mouth")).

Study selection

Study selection was performed independently by two reviewers. In the first stage, titles and abstracts were screened to identify studies involving periodontal regenerative therapy in human periodontal intrabony defects. Studies were excluded at this stage if they clearly did not involve periodontal intrabony defects, did not evaluate platelet-concentrate adjuncts, were not randomized controlled trials, were not conducted in humans, or were reviews, case reports, animal studies, or non-English publications.

The study selection process was guided by a predefined PICO framework. The population included adult patients with periodontal intrabony defects requiring regenerative periodontal therapy. The intervention was periodontal regenerative treatment using an autologous platelet-concentrate adjunct, including platelet-rich fibrin, concentrated growth factors, injectable platelet-rich fibrin, advanced platelet-rich fibrin, titanium-prepared platelet-rich fibrin, or related platelet-concentrate preparations used alone or with grafting materials. The comparator was regenerative periodontal therapy without a platelet-concentrate adjunct, including open-flap debridement, graft-only therapy, guided tissue regeneration, or another non-platelet-concentrate regenerative control. The outcomes included clinical and radiographic periodontal regeneration measures.

The primary outcome was clinical attachment level or relative attachment level gain, measured in millimeters. Secondary outcomes included probing pocket depth reduction, radiographic bone-fill or radiographic defect-depth reduction, gingival recession changes, and, when reported, cone-beam computed tomography-based bone measurements or biologic markers related to periodontal healing. For the quantitative synthesis, the main meta-analysis focused on clinical attachment level or relative attachment level gain, while a secondary radiographic analysis evaluated bone-fill or defect-depth reduction.

In the second stage, the full texts of potentially eligible articles were reviewed independently by the same two reviewers. Final eligibility was based on study design, intervention type, comparator group, clinical or radiographic periodontal outcomes, follow-up reporting, and availability of data suitable for qualitative or quantitative synthesis. Disagreements between reviewers were resolved by discussion and consensus. When consensus could not be reached, a third reviewer was consulted to make the final decision.

When studies included multiple intervention or control arms, the comparison most closely matching the review question was selected. The experimental arm was defined as platelet-concentrate adjunct regeneration (PCA), and the control arm was defined as regenerative therapy without a platelet-concentrate adjunct. This approach was used to maintain one consistent pairwise comparison across the meta-analysis and to avoid double-counting participants.

Data extraction

It was performed independently by two reviewers using a standardized extraction form. Extracted items included author name, publication year, study design, sample size, periodontal defect type, intervention, comparator, follow-up duration, clinical outcomes, radiographic outcomes, and numerical values required for meta-analysis. The main quantitative variables extracted were group mean, standard deviation, and total sample size for the experimental and control groups. Extracted data were compared between reviewers, and discrepancies were resolved through discussion. If disagreement persisted, a third reviewer reviewed the original article and made the final decision.

Data were extracted for study identification, intervention type, control treatment, sample size, clinical outcome measures, radiographic outcome measures, follow-up period, and numeric values required for meta-analysis. For the forest plots, the extracted variables included the mean, standard deviation, and total sample size for the experimental and control groups. The experimental group was standardized as platelet-concentrate adjunct regeneration, and the control group was standardized as regeneration without a platelet-concentrate adjunct. The main clinical analysis used clinical attachment level or relative attachment level gain in millimeters, and the secondary radiographic analysis used radiographic bone-fill or radiographic defect-depth reduction in millimeters.

When values were not presented in a RevMan-ready format, they were calculated only from data explicitly reported in the original articles, including group means, standard deviations, standard errors, confidence intervals (CIs), p-values, sample sizes, or change-from-baseline values when available. Standard errors and confidence intervals were converted to standard deviations using standard Cochrane methods when sufficient information was provided. If the required mean, standard deviation, or sample size could not be directly extracted or reproducibly calculated from the published article, that outcome was not entered into the quantitative synthesis. No unsupported substituted or assumed numeric values were used in the meta-analysis. Study authors were not contacted for missing data; therefore, studies or outcomes without extractable or reproducibly calculable quantitative data were excluded from the quantitative synthesis.

Data synthesis

The quantitative synthesis was structured around three forest plots. The first and main forest plot included all 10 selected randomized controlled trials and analyzed clinical attachment level or relative attachment level gain in millimeters [1-10]. This analysis was designed to provide the broadest estimate of the clinical regenerative effect of platelet-concentrate adjuncts. The second forest plot included 8 of the 10 studies and also focused on clinical attachment level or relative attachment level gain. This analysis served as a sensitivity-style comparison to evaluate whether the overall direction of effect remained consistent when fewer studies were pooled. The third forest plot included five studies and used radiographic bone-fill or radiographic defect-depth reduction as the outcome. This third analysis was intentionally different from the clinical attachment analysis because it assessed structural bone improvement rather than soft-tissue attachment gain. Together, the three forest plots allowed the review to examine both clinical and radiographic dimensions of periodontal regeneration (Table 2).

**Table 2:** Characteristics of included studies evaluating platelet-concentrate adjuncts for periodontal regeneration in intrabony defects A-PRF: advanced platelet-rich fibrin, beta-TCP: beta-tricalcium phosphate, BPBM: bovine porous bone mineral, CAL: clinical attachment level, CBCT: cone-beam computed tomography, CGF: concentrated growth factor, CT: computed tomography, DFDBA: demineralized freeze-dried bone allograft, FMBS: full-mouth bleeding score, FMPS: full-mouth plaque score, GI: gingival index, GRD: gingival recession depth, I-PRF: injectable platelet-rich fibrin, nHA: nanohydroxyapatite, OFD: open-flap debridement, PD: probing depth, PPD: probing pocket depth, PRF: platelet-rich fibrin, RAL: relative attachment level, RCT: randomized controlled trial, REC: relative position of gingival margin, RLDD: radiographic linear defect depth, T-PRF: titanium-prepared platelet-rich fibrin, VEGF: vascular endothelial growth factor

Study (reference)	Design	Sample size	Follow-up	Key outcomes reported
Qiao et al. (2016) [1]	Randomized controlled clinical trial	N=31 defects in 17 patients (CGF+BPBM=15; BPBM=16)	Baseline and 12 months	PPD reduction, CAL gain, radiographic defect-depth reduction, and bone fill comparing CGF+BPBM versus BPBM alone
Naqvi et al. (2017) [2]	Randomized double-blind split-mouth clinical trial	N=10 patients; 20 defects (PRF+bioactive glass putty=10; bioactive glass putty=10)	Baseline, 3, 6, and 9 months	Plaque index, gingival index, PPD reduction, CAL gain, and radiographic bone fill after treatment with PRF plus bioactive glass putty versus bioactive glass putty alone
Atchuta et al. (2020) [3]	Randomized three-arm clinical and radiographic trial	N=39 sites (OFD=13; DFDBA=13; DFDBA+PRF=13)	Baseline, 3, and 6 months	PPD, RAL, and radiographic bone fill comparing OFD, DFDBA alone, and DFDBA combined with PRF
Goyal et al. (2020) [4]	Randomized split-mouth clinical trial with high-resolution CT volumetric analysis	N=12 patients with paired defects; 24 sites (PepGen P-15=12; PepGen P-15+PRF=12)	Baseline, 3, and 6 months	PPD, RAL, REC, linear bone growth, and volumetric bone gain using CT-based assessment of PepGen P-15 with or without PRF
Bahammam and Attia (2021) [5]	Randomized controlled four-arm clinical trial	N=60 patients/sites (PRF=15; nano-HA=15; PRF+nano-HA=15; OFD=15)	Clinical/radiographic follow-up at 6 months; VEGF assessed early postoperatively	PD, GI, CAL, radiographic bone density, defect fill, and VEGF concentration after PRF, nano-HA, combined PRF+nano-HA, or OFD
Pavani et al. (2021) [6]	Randomized clinical trial with CBCT assessment	N=30 intrabony defect sites (OFD=10; beta-TCP+PRF=10; beta-TCP=10)	Baseline and 6 months	Plaque index, gingival index, sulcus bleeding index, PPD, CBCT defect-depth measurements, and bone fill after beta-TCP with or without PRF
Hazari et al. (2021) [7]	Randomized controlled clinical and radiographic trial	N=20 patients (Novabone putty=10; Novabone putty+PRF=10)	Baseline, 3, and 6 months	Plaque index, gingival index, PPD, RAL, and intraoral periapical radiographic defect-depth change comparing Novabone putty with or without PRF
Mallappa et al. (2022)	Randomized controlled clinicoradiographic trial	N=28 sites (A-PRF block+nHA=14; nHA=14)	Baseline and 6 months	Plaque index, gingival index, PPD, RAL, and radiographic linear defect-depth changes after A-PRF block with nHA versus nHA alone
Alshoiby et al. (2023) [9]	Double-blind randomized parallel-arm clinical trial	N=20 participants/defects (I-PRF+DFDBA=10; DFDBA=10)	Baseline, 3, 6, and 9 months	CAL, PPD, GRD, FMPS, FMBS, RLDD, and bone fill comparing I-PRF+DFDBA with DFDBA alone in stage-III periodontitis
Ozkal Eminoglu et al. (2024) [10]	Randomized controlled split-mouth clinical study	N=20 patients with 40 bilateral defects (OFD+T-PRF=20; OFD=20)	Baseline and 9 months; growth factors measured up to 12 weeks	Clinical, radiographic, and biochemical periodontal regeneration outcomes, including PPD, clinical endpoint, bone-filling rate, and growth factor levels

Risk of bias

Risk of bias was assessed independently by two reviewers using the Cochrane Collaboration risk-of-bias framework for randomized trials [27]. The domains assessed included random sequence generation, allocation concealment, blinding of participants and personnel, blinding of outcome assessment, incomplete outcome data, selective reporting, other sources of bias, and overall judgment. Each domain was rated as low risk, some concerns, or high risk based on the information reported in the original studies.

Because periodontal regenerative trials commonly involve surgical interventions, complete blinding of operators and participants was often difficult or not feasible. Therefore, particular attention was given to whether outcome assessors were blinded, whether randomization and allocation concealment were adequately described, whether follow-up data were complete, and whether all prespecified or clinically relevant outcomes were reported. Disagreements in risk-of-bias judgments were resolved through reviewer discussion and consensus. If consensus could not be reached, a third reviewer adjudicated the final judgment. The results of the risk-of-bias assessment are summarized in Table 3. Table 3 presents the findings from the risk-of-bias evaluation, conducted using the Cochrane tool (London, United Kingdom), for the studies that were included.

**Table 3:** Cochrane Collaboration's risk-of-bias assessment A-PRF: advanced platelet-rich fibrin, CBCT: cone-beam computed tomography, CGF: concentrated growth factors, DFDBA: demineralized freeze-dried bone allograft, HRCT: high-resolution computed tomography, i-PRF: injectable platelet-rich fibrin, PRF: platelet-rich fibrin, RCT: randomized controlled trial, T-PRF: titanium-prepared platelet-rich fibrin, VEGF: vascular endothelial growth factor Judgment categories: Low, Some concerns, and High were used to summarize the expected direction and magnitude of bias within each Cochrane risk-of-bias domain.

Study (year)	Random sequence generation	Allocation concealment	Blinding of participants/personnel	Blinding of outcome assessment	Incomplete outcome data	Selective reporting	Other bias	Overall judgment	Notes (concise rationale)
Qiao et al. (2016) [1]	Some concerns	Some concerns	Some concerns	Some concerns	Low	Low	Some concerns	Some concerns	Randomized intrabony-defect trial; allocation and examiner masking were not fully explicit; periodontal and radiographic outcomes were reported with minimal missing data.
Naqvi et al. (2017) [2]	Some concerns	Some concerns	Some concerns	Some concerns	Low	Low	Some concerns	Some concerns	Randomized controlled trial with small sample; surgical blinding was limited; clinical/radiographic outcomes were reported and attrition appeared low.
Atchuta et al. (2020) [3]	Some concerns	Some concerns	Some concerns	Some concerns	Low	Low	Some concerns	Some concerns	Three-arm randomized clinical study; selected comparison matched PRF plus graft versus non-platelet adjunct regeneration; blinding details were limited but outcome reporting was complete.
Goyal et al. (2020) [4]	Some concerns	Some concerns	Some concerns	Low	Low	Low	Some concerns	Some concerns	Randomized split-mouth design with objective HRCT-assisted volumetric assessment; participant/operator blinding was not feasible, but outcome measurement was largely objective.
Bahammam and Attia (2021) [5]	Low	Some concerns	Some concerns	Low	Low	Low	Some concerns	Some concerns	Randomized controlled design with clinical, radiographic, and VEGF-related outcomes; allocation concealment details were not fully clear; missing data were limited.
Pavani et al. (2021) [6]	Some concerns	Some concerns	Some concerns	Low	Low	Low	Some concerns	Some concerns	Randomized clinical trial using CBCT for bone fill; examiner/objective imaging assessment strengthened detection bias domain; surgical blinding remained limited.
Hazari et al. (2021) [7]	Some concerns	Some concerns	Some concerns	Some concerns	Low	Low	Some concerns	Some concerns	Randomized control trial comparing NovaBone Putty with and without PRF; blinding and concealment were not fully described; reported outcomes were clinically relevant and complete.
Mallappa et al. (2022) [8]	Low	Some concerns	Some concerns	Low	Low	Low	Some concerns	Some concerns	Randomized clinicoradiographic study using A-PRF block and CBCT assessment; outcome evaluation was objective, although allocation concealment and surgical blinding were limited.
Alshoiby et al. (2023) [9]	Low	Some concerns	Some concerns	Low	Low	Low	Some concerns	Some concerns	Randomized controlled clinical trial comparing i-PRF plus DFDBA versus DFDBA; periodontal outcomes were clearly reported; concealment and performance blinding required cautious interpretation.
Ozkal Eminoglu et al. (2024) [10]	Low	Low	Some concerns	Low	Low	Low	Low	Some concerns	Randomized split-mouth clinical study; objective clinical, biochemical, and radiographic measures were reported; performance blinding was limited due to the surgical intervention.

Statistical analysis

Because the included randomized controlled trials used both parallel-arm and split-mouth designs, the handling of paired split-mouth data was considered during the quantitative synthesis. A formal split-mouth correlation adjustment was not performed because the included split-mouth trials did not consistently report the within-patient correlation coefficient, paired-difference standard deviation, or other paired-analysis parameters required for a reproducible paired-data calculation. Therefore, split-mouth trials were pooled using available study-level summary data, including group means, standard deviations, and sample sizes. This approach represents a major methodological limitation of the meta-analysis because treating paired split-mouth data as independent parallel-group data may affect the precision of the effect estimates and confidence intervals. For this reason, the pooled results should be interpreted cautiously, particularly for analyses containing split-mouth trials.

The 10-study clinical attachment level or relative attachment level forest plot showed an overall mean difference favoring platelet-concentrate adjunct regeneration, with low heterogeneity. The 8-study clinical forest plot showed a similar favorable direction of effect, also with low heterogeneity. The 5-study radiographic forest plot showed a favorable direction of effect for platelet-concentrate adjunct regeneration on radiographic bone-fill or defect-depth reduction, but the result did not reach statistical significance and substantial heterogeneity was detected. These findings suggest that the clinical analyses were directionally consistent, while the radiographic analysis should be interpreted more cautiously because of its wider confidence interval and substantial heterogeneity. Review Manager version 5.4 was used to structure the meta-analysis approach and generate forest plots. A funnel plot was also used to visually assess potential small-study effects and publication bias in the main clinical dataset. The funnel plot showed that most studies were clustered around the pooled estimate, although some asymmetry was present. Because the number of included studies was limited, the funnel plot was interpreted cautiously rather than as definitive evidence for or against publication bias.

Literature search and study selection

**Figure 1:**
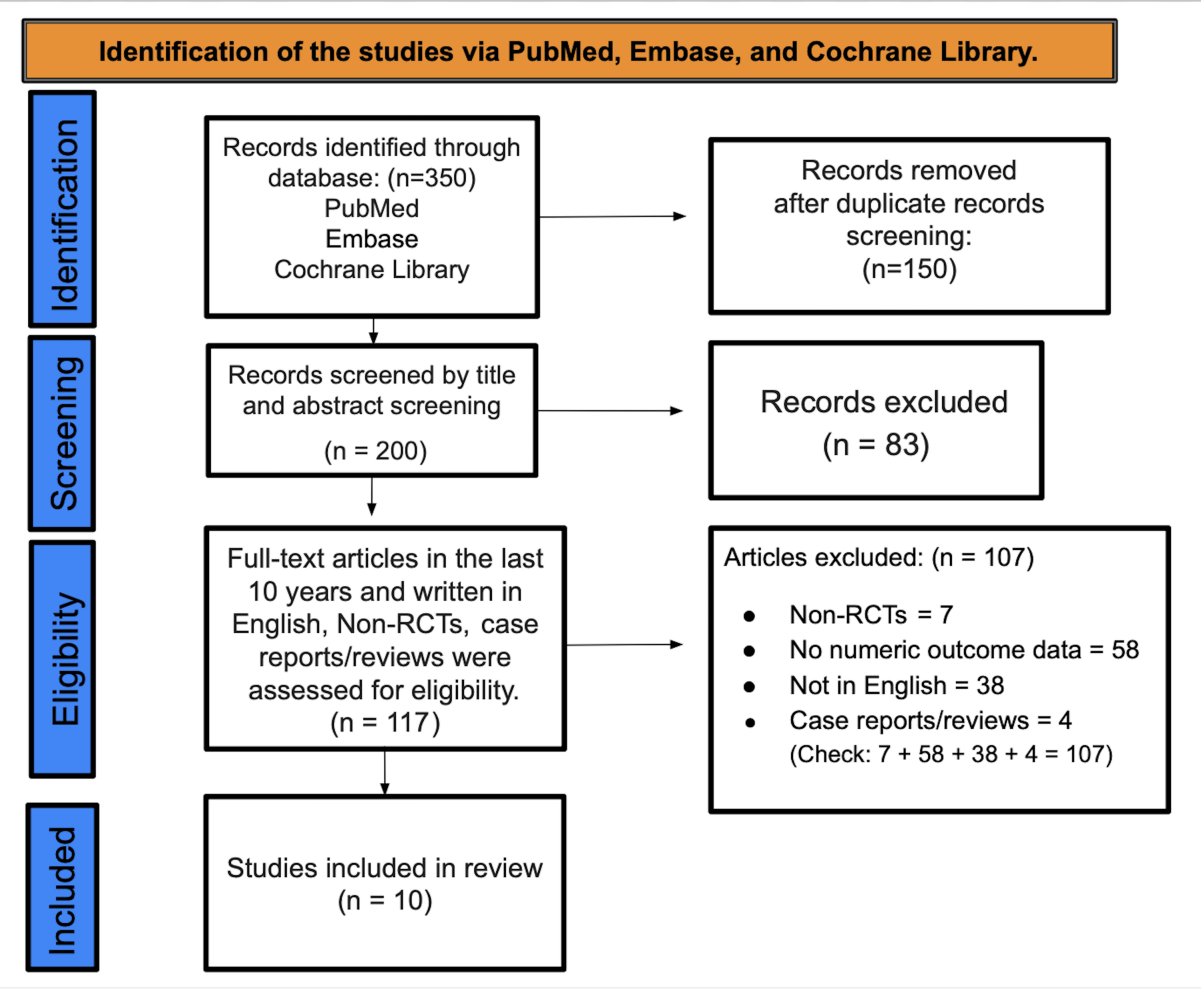
PRISMA diagram PRISMA: Preferred Reporting Items for Systematic Reviews and Meta-Analyses, RCT: randomized controlled trial

Researchers initially found 350 articles across PubMed, Embase, and Cochrane databases. After removing 150 duplicate entries, 200 records remained for title and abstract review, which led to the exclusion of 83 studies. The remaining 117 articles underwent full-text assessment using specific criteria: publication within the past 10 years, written in English, randomized controlled trial design, and accessible outcome data. Of these, 107 were excluded: 7 were not randomized controlled trials, 58 lacked quantitative data, 38 were not in English, and 4 were case reports or reviews. Ultimately, 10 studies met all requirements and were included in the qualitative analysis (Figure 1).

Evidence synthesis: Principal outcomes and implications for practice

Between-study heterogeneity was evaluated using Cochrane's χ² test and quantified with I², while pooled treatment effects were calculated in RevMan 5.4 using the inverse-variance random-effects model.

**Figure 2:**
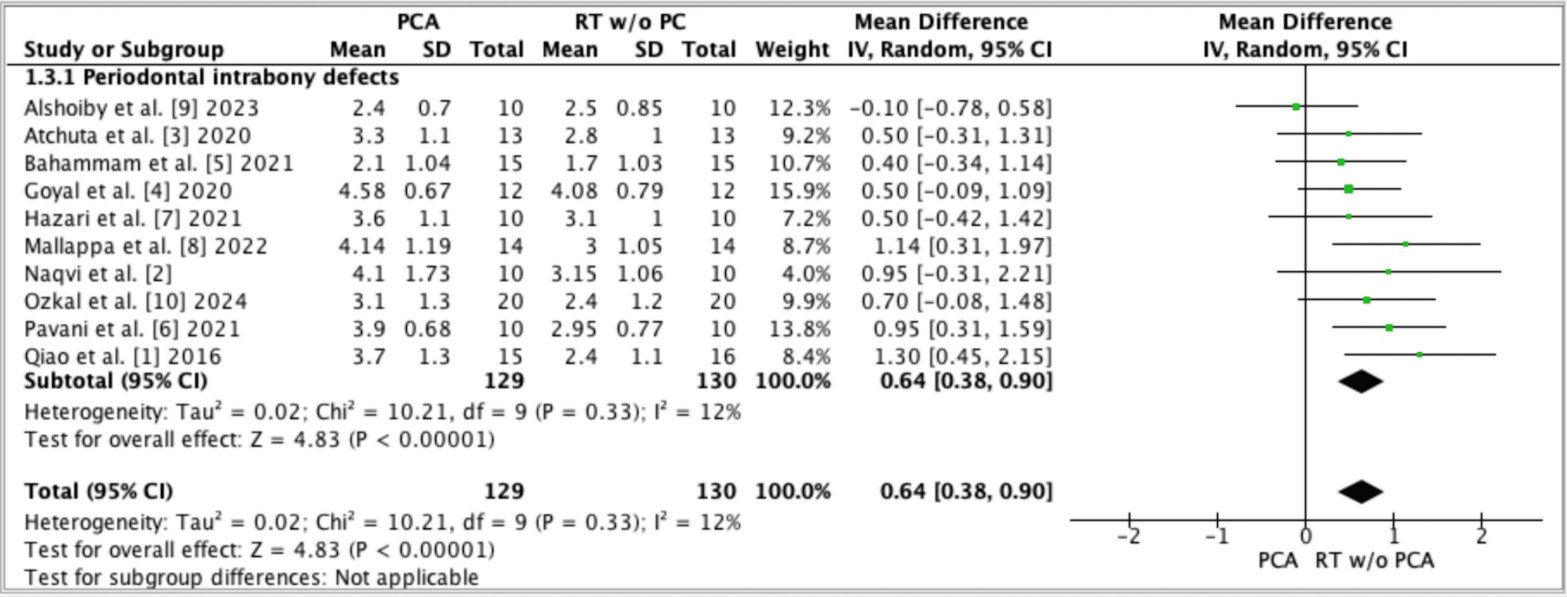
Forest plot of the overall clinical effect of platelet-concentrate adjuncts in periodontal intrabony defects Forest plot comparing PCA with RT w/o PC for periodontal intrabony defects. This analysis included 10 randomized controlled trials and evaluated clinical attachment level or relative attachment level gain in millimeters. The outcome was analyzed as MD using a random-effects inverse-variance model. The pooled estimate favored PCA, indicating greater clinical attachment improvement in the experimental group compared with the control group (MD = 0.64 mm, 95% CI: 0.38 to 0.90; p < 0.00001). Heterogeneity was low (I² = 12%). Squares represent individual study effects, horizontal lines represent 95% CIs, and the diamond represents the pooled effect estimate. PCA: platelet-concentrate adjunct regeneration, RT w/o PC: regenerative therapy without platelet concentrate, MD: mean difference, CI: confidence interval, IV: inverse variance

The first meta-analysis presents the overall clinical meta-analysis of 10 randomized controlled trials comparing platelet-concentrate adjunct regeneration (PCA) with regenerative therapy without platelet concentrate (RT w/o PC) for periodontal intrabony defects. The pooled mean difference favored PCA, showing greater clinical attachment level or relative attachment level gain compared with control therapy (MD = 0.64 mm, 95% CI: 0.38 to 0.90; Z = 4.83; p < 0.00001). Heterogeneity was low (Tau² = 0.02; χ² = 10.21, df = 9; p = 0.33; I² = 12%), supporting consistency across the included trials (Figure 2).

**Figure 3:**
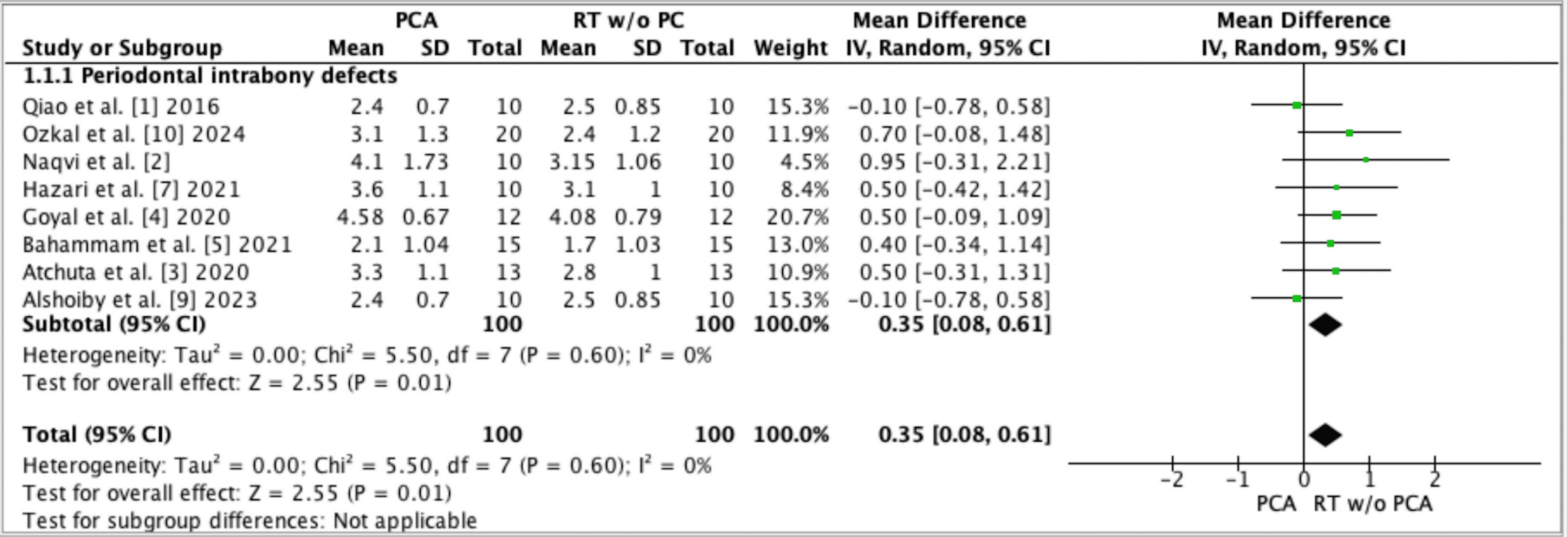
Forest plot of the sensitivity analysis using selected randomized controlled trials Forest plot of the reduced clinical sensitivity analysis comparing PCA with RT w/o PC for periodontal intrabony defects. This analysis included eight randomized controlled trials and evaluated clinical attachment level or relative attachment level gain in millimeters. The outcome was analyzed as MD using a random-effects inverse-variance model. The pooled estimate favored PCA, showing greater clinical attachment improvement compared with control therapy (MD = 0.35 mm, 95% CI: 0.08 to 0.61; p = 0.01). No important heterogeneity was detected (I² = 0%). Squares represent individual study effects, horizontal lines represent 95% CIs, and the diamond represents the pooled effect estimate. PCA: platelet-concentrate adjunct regeneration, RT w/o PC: regenerative therapy without platelet concentrate, MD: mean difference, CI: confidence interval, IV: inverse variance

The second meta-analysis presents the reduced clinical sensitivity analysis, including eight randomized controlled trials that reported clinical attachment level or relative attachment level gain. The pooled effect remained in favor of PCA, although the magnitude of effect was smaller than in the full 10-study analysis (MD = 0.35 mm, 95% CI: 0.08 to 0.61; Z = 2.55; p = 0.01). No important statistical heterogeneity was detected in this analysis (Tau² = 0.00; χ² = 5.50, df = 7; p = 0.60; I² = 0%). These findings suggest that the favorable clinical effect of PCA remained directionally stable when the dataset was restricted to selected studies (Figure 3).

**Figure 4:**
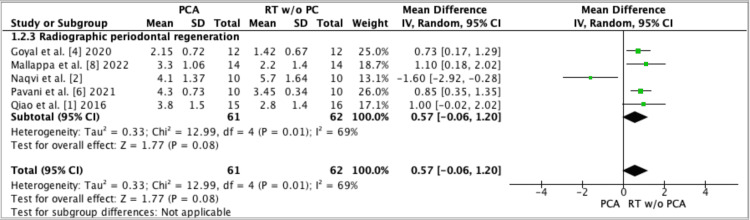
Forest plot of radiographic periodontal regeneration outcomes Forest plot comparing PCA with RT w/o PC for radiographic periodontal regeneration in intrabony defects. This analysis included five studies and evaluated radiographic bone fill or radiographic defect-depth reduction in millimeters. The outcome was analyzed as MD using a random-effects inverse-variance model. The pooled mean difference favored PCA; however, the CI crossed the line of no effect, and the result did not reach conventional statistical significance (MD = 0.57 mm, 95% CI: -0.06 to 1.20; p = 0.08). Heterogeneity was substantial (I² = 69%), suggesting variability in radiographic measurement methods, platelet-concentrate protocols, grafting materials, and follow-up periods. Squares represent individual study effects, horizontal lines represent 95% CIs, and the diamond represents the pooled effect estimate. PCA: platelet-concentrate adjunct regeneration, RT w/o PC: regenerative therapy without platelet concentrate, MD: mean difference, CI: confidence interval, IV: inverse variance

The third meta-analysis evaluated radiographic periodontal regeneration using bone-fill or defect-depth reduction outcomes from five studies. The pooled mean difference also favored PCA; however, the confidence interval crossed the line of no effect, and the overall result did not reach conventional statistical significance (MD = 0.57 mm, 95% CI: -0.06 to 1.20; Z = 1.77; p = 0.08). Heterogeneity was substantial (Tau² = 0.33; χ² = 12.99, df = 4; p = 0.01; I² = 69%), indicating greater variability among radiographic outcomes. This variability may reflect differences in imaging methods, defect morphology, grafting materials, platelet-concentrate protocols, and follow-up duration across the included studies (Figure 4).

**Figure 5:**
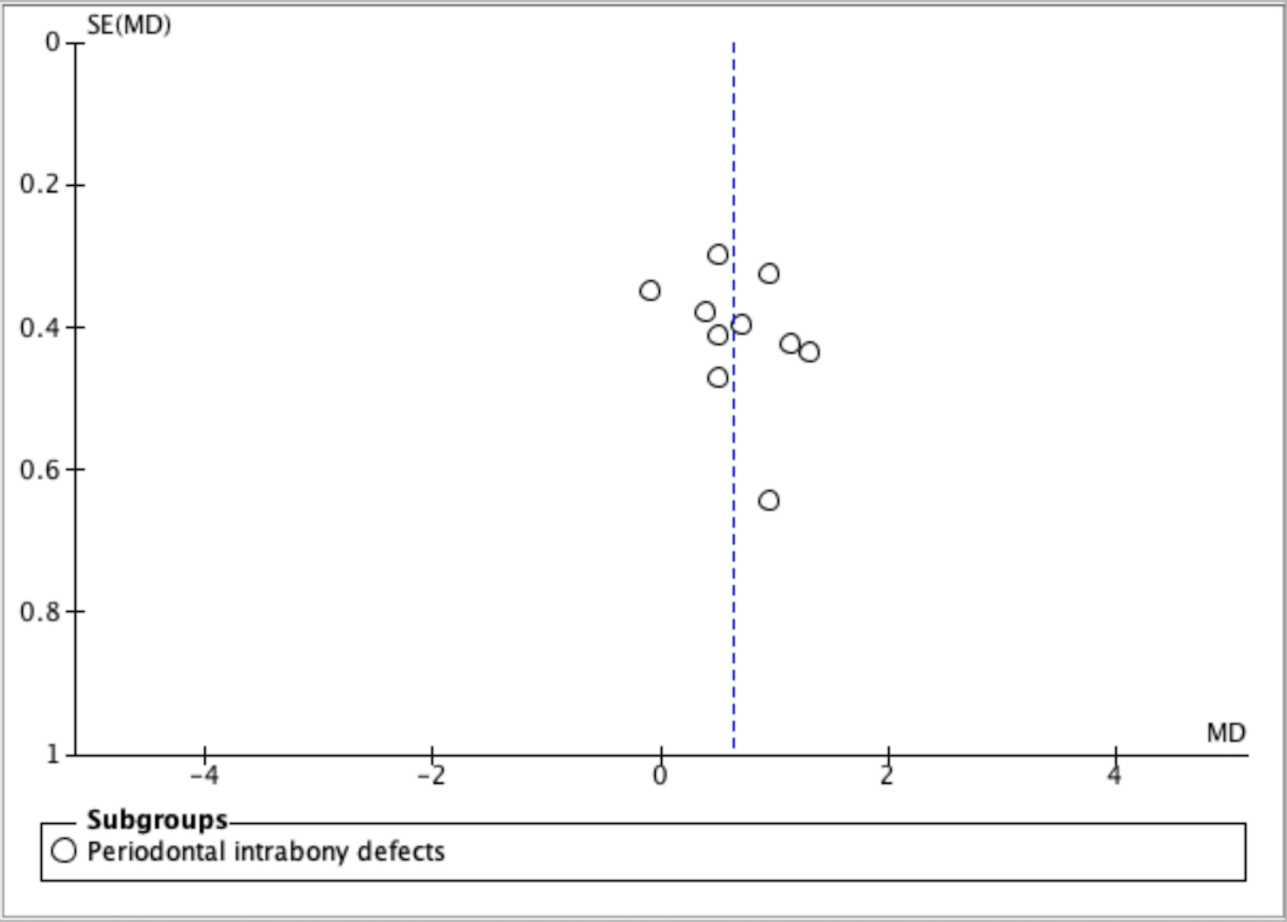
Funnel plot assessing publication bias Funnel plot evaluating potential small-study effects or publication bias among the included studies. The horizontal axis represents the mean difference, and the vertical axis represents the standard error of the mean difference. Each circle represents one study. Visual inspection shows that most studies were distributed near the pooled estimate, suggesting no strong evidence of major publication bias; however, interpretation should remain cautious because funnel plots are less reliable when the number of included studies is limited.

A funnel plot was used as a visual method to explore possible small-study effects or publication bias in the main clinical meta-analysis. In this plot, each point represents one included study, with the treatment effect shown on the horizontal axis and the standard error shown on the vertical axis. Studies with smaller standard errors appear toward the top of the plot, while smaller studies with larger standard errors appear toward the bottom. Most studies were located near the pooled mean difference, and no clear pattern of major asymmetry was observed. This suggests that there was no strong visual evidence of publication bias. However, because the number of included studies was limited, the funnel plot was interpreted cautiously and was not considered definitive evidence for confirming or excluding publication bias (Figure 5).

Discussion

This systematic review and meta-analysis suggests that autologous platelet-concentrate adjuncts may provide additional regenerative benefit when used in the surgical management of periodontal intrabony defects. Across the selected randomized controlled trials, the direction of effect generally favored platelet-concentrate adjunct regeneration over regenerative therapy without platelet concentrates, especially for clinical attachment or relative attachment gain and radiographic bone-fill outcomes [1-10]. This finding is biologically plausible because platelet-rich fibrin, concentrated growth factors, injectable platelet-rich fibrin, advanced platelet-rich fibrin, and titanium-prepared platelet-rich fibrin can act as autologous fibrin scaffolds that support clot stability, growth factor release, angiogenesis, and early wound maturation [1,6,8-10]. In periodontal intrabony defects, where regeneration depends on stable wound healing and repopulation by periodontal ligament-derived cells, these biologic properties may help explain why several trials reported favorable clinical and radiographic improvements when platelet concentrates were combined with grafting materials or open-flap debridement [2,4,5,7,9].

The overall 10-study clinical forest plot shown in Figure 2 demonstrated a statistically significant improvement in clinical attachment level or relative attachment level gain favoring platelet-concentrate adjunct regeneration. The reduced eight-study clinical sensitivity analysis shown in Figure 3 supported the same favorable direction of effect. The five-study radiographic analysis shown in Figure 4 also favored platelet-concentrate adjunct regeneration for bone-fill or defect-depth reduction, although the result did not reach statistical significance and showed substantial heterogeneity. Therefore, the clinical findings appear more consistent than the radiographic findings, which should be interpreted more cautiously.

The clinical significance of these pooled mean differences should be interpreted in relation to the incremental nature of periodontal regenerative therapy. In the main 10-study clinical analysis, the pooled mean difference of 0.64 mm in clinical attachment level or relative attachment level gain suggests a modest but potentially meaningful additional benefit when platelet-concentrate adjuncts are added to regenerative therapy. Although a mean difference of less than 1 mm should not be interpreted as a large stand-alone clinical effect, even small improvements in attachment gain may be relevant in periodontal intrabony defects when they occur together with probing-depth reduction, radiographic bone improvement, improved wound stability, and long-term defect stabilization. Therefore, the observed benefit is best understood as an adjunctive improvement rather than a replacement for proper surgical technique, defect selection, plaque control, and maintenance therapy.

The reduced eight-study clinical sensitivity analysis showed a smaller pooled benefit of 0.35 mm, which supports the same favorable direction of effect but suggests that the magnitude of clinical improvement may be limited when more selected studies are analyzed. This effect size may be considered modest in routine periodontal practice and may not be clinically important for every patient or defect. However, in deep or anatomically favorable intrabony defects, a small additional gain may still contribute to improved periodontal stability when combined with other regenerative outcomes.

For the radiographic analysis, the pooled mean difference of 0.57 mm favored platelet-concentrate adjunct regeneration, but the confidence interval crossed the line of no effect, and the result did not reach statistical significance. Therefore, the radiographic finding should be interpreted cautiously. It suggests a possible structural benefit in bone-fill or defect-depth reduction, but the clinical importance of this effect remains uncertain because of substantial heterogeneity and variation in imaging methods, grafting materials, platelet-concentrate protocols, and follow-up periods. Overall, the findings suggest that platelet-concentrate adjuncts may provide clinically useful but modest additional benefit, especially for clinical attachment outcomes, while the radiographic benefit requires more cautious interpretation and confirmation in larger standardized trials.

The consistency between the main and sensitivity clinical analyses suggests that the observed clinical benefit was not driven by one isolated study or one specific platelet-concentrate preparation. However, the radiographic findings were less certain because of the smaller number of included studies, wider confidence interval, and greater heterogeneity.

A key strength of this review is the focused clinical question. Instead of combining different periodontal indications, the review concentrated on periodontal intrabony defects, which are among the most appropriate defect types for regenerative therapy [1-10]. This focus improves clinical comparability because most included trials evaluated similar outcomes, such as probing pocket depth reduction, clinical attachment gain, relative attachment gain, and radiographic bone fill [2,3,5,7,10]. The additional related studies also support the relevance of this topic because several randomized trials have explored platelet-rich fibrin with demineralized freeze-dried bone allograft, enamel matrix derivative, hydroxyapatite, guided tissue regeneration, and other regenerative materials in comparable periodontal defects [16-24]. Together, these studies suggest that platelet concentrates are not being investigated as isolated products, but as part of a broader regenerative strategy that depends on biologic stimulation, scaffold support, defect morphology, and surgical technique [18,20,23,25-27].

Despite these encouraging findings, several limitations must be considered. The included studies were heterogeneous in platelet-concentrate preparation protocols, centrifugation methods, grafting materials, follow-up duration, defect selection, and outcome measurement [1-10]. For example, some trials evaluated platelet concentrates with bone substitutes, while others used open-flap debridement or biologic materials as the comparator [1,2,5,7,9]. This variation makes the pooled effect clinically informative, but not perfectly uniform, because the observed benefit may reflect the combined influence of the platelet concentrate, graft material, and surgical environment [3,4,6,8,10]. In addition, some studies had relatively small sample sizes or split-mouth designs, which can increase imprecision and may overstate treatment effects when methodological safeguards are incomplete [16,17,21,24].

Risk of bias is another important issue. Surgical periodontal trials are difficult to blind because the operator usually knows which regenerative material is being applied, and participant blinding may also be limited by the surgical protocol [1-10]. Therefore, performance bias is a common concern in this type of research, even when the outcomes are measured objectively [16,19,22,25]. The most important safeguards are adequate random sequence generation, allocation concealment, examiner blinding, complete follow-up, and transparent outcome reporting [3,6,9,18,27]. When these elements are unclear, the certainty of the evidence decreases, even if the numerical result favors the intervention [2,5,8,10,23]. This is especially relevant for this review because some forest plot values required reproducible calculation from data explicitly reported in the original articles when RevMan-ready means and standard deviations were not directly presented. Outcomes without sufficient extractable or calculable data were not included in the quantitative synthesis, and the pooled results should therefore be interpreted with attention to the quality and completeness of the original reporting [1-10].

Another limitation is the combined inclusion of parallel-arm and split-mouth randomized controlled trials. Although split-mouth designs are common in periodontal regeneration research, several included split-mouth studies did not provide the within-patient correlation coefficients or paired-difference standard deviations needed for formal paired-data adjustment. As a result, split-mouth correlation adjustment was not performed in the primary meta-analysis. This may have influenced the precision of the effect sizes, and the pooled findings should therefore be interpreted with this methodological limitation in mind.

The funnel plot shown in Figure 5 also requires careful interpretation. Although the plotted studies appeared to cluster around the pooled effect, visual asymmetry cannot be ruled out because the number of included studies was limited [1-10]. In small meta-analyses, funnel plots have low ability to distinguish true publication bias from chance, clinical heterogeneity, selective outcome reporting, or small-study effects [16,20,24]. Therefore, the funnel plot should be considered a supportive visual tool rather than conclusive evidence that publication bias is absent [1-10,16-25]. Future reviews would benefit from including additional high-quality trials with prospectively registered protocols, standardized outcome reporting, and accessible full datasets [21,25,27].

Overall, this systematic review and meta-analysis supports the potential role of autologous platelet concentrates as useful adjuncts for periodontal regeneration in intrabony defects [1-10]. The findings suggest that platelet-concentrate adjuncts may improve clinical attachment outcomes and may support radiographic evidence of bone regeneration when compared with similar regenerative therapy without platelet concentrates [1,2,4,6,8-10]. Nevertheless, the strength of the conclusion is moderated by heterogeneity in protocols, small sample sizes, incomplete reporting of some numeric outcomes, lack of formal split-mouth correlation adjustment, and unavoidable challenges in blinding surgical interventions [16-18,22,24,27]. Clinically, platelet concentrates appear promising because they are autologous, biologically active, and compatible with multiple regenerative materials, but they should be viewed as adjuncts rather than replacements for proper case selection, surgical technique, plaque control, and maintenance therapy [5,7,9,20,25,26]. Future randomized controlled trials should use standardized preparation protocols, longer follow-up, calibrated blinded examiners, appropriate handling of split-mouth data, and consistent reporting of clinical and radiographic outcomes to clarify the true magnitude of benefit in periodontal regeneration [1-10,16-25].

Limitations

A major limitation of this meta-analysis is the combined inclusion of parallel-arm and split-mouth randomized controlled trials without formal paired-data adjustment for the split-mouth studies. Several split-mouth trials did not report the within-patient correlation coefficient or paired-difference standard deviation required for reproducible correlation-adjusted analysis. As a result, these studies were analyzed using available group-level summary data. This may have influenced the precision of the pooled estimates and confidence intervals. Nevertheless, this approach avoided the use of unsupported correlation assumptions and allowed the available randomized evidence to be synthesized transparently. Future trials should report paired-effect estimates or the statistical parameters needed for appropriate split-mouth meta-analysis.

Additional limitations should also be considered when interpreting the findings. Although the included studies were randomized controlled trials and focused on periodontal intrabony defect regeneration, there was some variation in platelet-concentrate preparation, grafting materials, surgical protocols, follow-up periods, and outcome-reporting methods. These differences may have introduced clinical and methodological heterogeneity across studies. In addition, some trials had relatively small sample sizes, which is common in surgical periodontal research. However, the overall direction of effect remained consistent across the clinical analyses, supporting the stability of the main findings. Future studies with larger samples, standardized platelet-concentrate protocols, appropriate split-mouth statistical reporting, and longer follow-up periods would help strengthen the evidence further.

Another consideration is the presence of clinical heterogeneity across the included trials. Although all studies focused on periodontal intrabony defects and evaluated platelet-concentrate adjuncts, there were differences in defect characteristics, platelet-concentrate preparation protocols, grafting materials, surgical approaches, imaging methods, and follow-up periods. These factors may have contributed to variation in the magnitude of clinical and radiographic effects. However, the overall direction of the findings remained generally favorable across the main and sensitivity analyses, suggesting that the results were reasonably consistent despite these expected differences in periodontal surgical research. In addition, a formal GRADE certainty-of-evidence assessment was not performed, which should be considered when interpreting the strength of the conclusions. Nevertheless, the interpretation of the findings considered key certainty-related factors, including study design, risk of bias, consistency of effect, precision, and heterogeneity.

Protocol registration statement

This systematic review and meta-analysis was not prospectively registered in PROSPERO or another international review registry. This should be acknowledged as a methodological limitation because prospective registration improves transparency and allows readers to verify whether the final review methods followed the original protocol. However, before completing the final analysis, the review question, eligibility criteria, included outcomes, data extraction approach, risk-of-bias domains, and statistical methods were defined and applied consistently across the included randomized controlled trials. No patient-level data were collected, and the study was based exclusively on previously published evidence. Therefore, although the absence of prospective registration may limit protocol transparency, it is unlikely to have altered the overall direction of the findings. Future updates of this review should be prospectively registered before study selection and data extraction.

Ethics statement

This study is a systematic review and meta-analysis based exclusively on data extracted from previously published studies. No new human participants were recruited, no direct patient contact occurred, and no identifiable private information was collected or analyzed. Therefore, institutional review board approval was not required. Informed consent was not applicable for this study.

## Conclusions

In conclusion, this systematic review and meta-analysis suggests that autologous platelet-concentrate adjuncts may provide a clinically useful benefit in regenerative periodontal therapy for intrabony defects. The pooled findings favored platelet-concentrate use for clinical attachment improvement and showed a supportive trend for radiographic bone regeneration. These results support their potential value as adjuncts in periodontal practice, while future standardized trials can further strengthen the evidence.
